# Clinician factors influencing decision-making in emergency general surgery (EGS): a scoping review protocol

**DOI:** 10.1136/bmjopen-2026-116205

**Published:** 2026-04-15

**Authors:** Ellen Groundwater, Carly Nichola Bisset, Rebecca Bradley, Rachel John-Charles, Yasuko Maeda, Tracey McKee, Susan Joan Moug

**Affiliations:** 1 Department of Surgery, NHS Greater Glasgow and Clyde, Royal Alexandra Hospital, Paisley, UK; 2 University of Glasgow, Glasgow, UK; 3 Department of Surgery, NHS Greater Glasgow and Clyde, Glasgow Royal Infirmary, Glasgow, UK; 4 Department of Surgery, Golden Jubilee National Hospital, Clydebank, UK; 5 Department of Surgery, NHS Greater Glasgow and Clyde, Queen Elizabeth University Hospital, Glasgow, UK; 6 Knowledge Services, NHS Greater Glasgow and Clyde, Glasgow, UK

**Keywords:** Adult surgery, SURGERY, Decision Making, Clinical Decision-Making

## Abstract

**Abstract:**

**Introduction:**

Emergency general surgery (EGS) decisions often occur under time pressure and with reduced patient involvement in comparison to the elective setting. Variation in decision-making in the field of EGS is partly attributable to patient and contextual factors, but clinician factors also likely shape decisions.

This scoping review aims to clarify which clinician factors have been investigated in relation to decision-making in adult EGS, which have been identified as influential, and the methods used to measure them.

**Methods and analysis:**

Any studies reporting on individual clinician factors and decision-making in EGS will be eligible. Studies must be only in adult populations (patients aged 18 and over). This review will consider quantitative, qualitative and mixed-methods study designs.

This scoping review will be conducted in accordance with the methodology developed by the Joanna Briggs Institute and reported in accordance with Preferred Reporting Items for Systematic Review and Meta-Analysis Protocols guidelines. This protocol was prospectively registered with the Open Science Framework: https://osf.io/xcqp4. We will search MEDLINE, EMBASE, CINAHL, CDSR and CENTRAL. The search strategy will be adapted for each database, and no time or language restrictions will be applied.

Two independent reviewers will screen studies for eligibility and extract data using a purpose-designed data extraction form, with disagreements resolved by a third reviewer. Extracted data will be synthesised using a narrative approach to map key concepts, describe study characteristics and identify gaps in the literature.

**Ethics and dissemination:**

Ethical approval is not required as this review will use publicly available data. Findings will be disseminated through peer-reviewed publication and conference presentations.

**Strengths and limitations of this study**

- This protocol follows methodological guidance from the Joanna Briggs Institute and is reported in accordance with Preferred Reporting Items for Systematic Review and Meta-Analysis Protocols guidelines.
- Quantitative, qualitative and mixed-methods studies will be included, allowing for a broad exploration of how clinician factors are conceptualised and measured.
- The existing literature examining clinician factors in emergency general surgery decision-making may be limited.
- Variation in definitions of emergency general surgery and clinician factors across studies and healthcare systems may affect comparability of findings.

## Introduction

 Emergency general surgery (EGS) is defined as ‘any patient requiring emergency surgical evaluation (operative or non-operative) for diseases within the realm of general surgery’.^1^ Some patients will require emergency surgical intervention for their presenting pathology, while others will be appropriately managed with medical therapy alone. Among patients whose pathology indicates the need for surgical intervention, two key groups can be identified: those who proceed to emergency laparotomy and those who do not, the NoLap population.^2^

EGS encompasses a vast array of surgical pathology and contributes to a significant proportion of admissions under the banner of general surgery.^3^ EGS also forms a substantial proportion of the mortality observed in surgical patients, with 80% of surgical mortality arising as a result of emergency surgical intervention.^4^ Understanding and optimising the decision-making process for EGS patients is crucial for improving patient outcomes and mortality rates. The decision-making in EGS often occurs under time pressure and uncertainty, which impacts the ability to engage in effective shared decision-making.^5^

In this review, we considered all clinicians involved in EGS decision-making. Clinicians involved in EGS encounter numerous decision-making points in the management of their patients and there remains a large variation in this decision-making.^6^ These decisions may include, but are not limited to, decisions regarding initial assessment and investigations, whether to pursue operative or non-operative management (including NoLap patients), the type of operation or anaesthetic, the timing or prioritisation of surgical intervention, decisions regarding escalation of care or referral, palliative management, in addition to intraoperative and postoperative management decisions.

The variation seen in decision-making is partly attributable to patient and contextual factors, but these factors alone do not account for the significant variation seen in current practice. It is likely that clinician factors also influence decision-making. A clinician factor is defined as ‘a trait or attribute related to a clinician which influences the assessment and/or treatment of a patient’.^7^

In this review, we considered clinician factors in three categories:

1. Demographic factors.
2. Training experience.
3. Psychosocial factors.

Demographic factors include a clinician’s gender, age and ethnicity, as well as their cultural background. In addition, the type of hospital in which a clinician practises may expose them to different cases and institutional cultures.^8^

The training experience of a clinician refers to the experience and expertise accumulated over their career. The career stage of the clinician reflects their level of experience within a specific medical field and encompasses medical students, junior doctors and consultants (or their international equivalents). Training factors also include a clinician’s specialty and sub-speciality. In the modern era of medicine, sub-specialisation is becoming ever more complex due to advancing medical knowledge and technologies.^9^ Clinicians involved in EGS will have varying degrees of their workload dedicated to the field, ranging from infrequent involvement on-call to dedicated EGS clinicians. This review seeks to establish whether clinician experience or expertise has been examined in relation to EGS decision-making.

Psychosocial factors are perhaps the least studied of these clinician factors. Psychosocial factors are the combined effects of individual psychology and the social environment on behaviour, attitudes and decision-making.^10^ This review aims to identify whether personality or individual personality traits of the clinicians involved in EGS decision-making have been studied.

Heuristics, which can be seen as mental shortcuts, can aid in making rapid decisions and are often employed in time-pressured situations in medicine. The use of heuristics can improve efficiency but introduces the potential for cognitive bias, which is unconscious and systematic errors in thinking that can influence decisions and judgement.^11^ This review will identify and synthesise the current evidence on heuristics and biases in EGS decision-making.

There is limited evidence examining clinician factors and their influence on decision-making in the realm of EGS. The current evidence spans multiple disciplines, including medical literature and psychological research, and is characterised by a wide variety of study designs and methods. This scoping review aims to map the current literature and identify gaps, with the hope of informing future study.

### Review question

Which clinician factors have been investigated in relation to decision-making in adult EGS, which have been identified as influential and by what methods are these factors measured?

### Objectives

1. To identify and map clinician factors studied in relation to decision-making in adult EGS.
2. To describe decision points and/or outcomes examined.
3. To summarise study designs, measures and analytical approaches.
4. To identify knowledge gaps and priorities for future research.

### Definition

#### Clinician factor

‘A trait or attribute related to a clinician which influences the assessment and/or treatment of a patient’.^7^

### Examples of clinician factors

Detailed below is a comprehensive, but not exhaustive, list of clinician factors.

#### Demographic factors

- Age.
- Sex (most often available), gender (if available).
- Country of practice.
- Country of study/training.
- Native language/language of clinical practice.
- Hospital setting (eg, teaching hospital, district general hospital).
- Work setting (eg, private practice, public sector, rural vs urban).
- Ethnicity.
- Cultural background/cultural influences.

#### Training

- Experience, career stage (eg, consultant/attending, registrar/resident, trainee, intern, student), level of training, grade of training, years of surgical experience.
- Sub-speciality (eg, general surgery, EGS, trauma surgery, colorectal surgery, upper gastrointestinal surgery, hepatobiliary surgery).

#### Psychosocial factors (with attached definitions)

- Personality/personal traits—defined as personal qualities and emotions that shape a person’s characteristic behaviour and thoughts.^12^
- Propensity to risk, risk-taking behaviour, risk tolerance, risk perception—these words describe an individual’s overall approach to uncertainty.^13^
- Self-awareness—defined as the ability of an individual to recognise their own emotions and/or behaviours and the subsequent effect on others.^14^
- Coping style—defined as strategies an individual can employ to manage stress.
- Cognition, cognitive bias, heuristics—these words describe mental processes involved in acquiring knowledge and making decisions.^11^
- Fatigue, stress, burnout—these words describe physical and/or emotional exhaustion which has resulted from persistent stressors.
- Attitude, behaviours, beliefs—these words describe the personal viewpoints that shape responses.
- Personal bias—defined as preconceived notions held by an individual.^15^

## Methods

The proposed scoping review will be conducted in accordance with the Joanna Briggs Institute (JBI) methodology for scoping reviews and is reported following the Preferred Reporting Items for Systematic Review and Meta-Analysis Protocols (PRISMA-P) guidelines.^16^,^18^

### Registration

This scoping review protocol was prospectively registered on the Open Science Framework: https://osf.io/xcqp4.

### Inclusion and exclusion criteria

The population/concept/context (PCC) framework, recommended by the JBI, has been employed in this review to identify the main concepts in the primary review question and construct the eligibility criteria. In this review, the PCC framework has been employed as such:

- Population—clinicians involved in adult EGS.
- Concept—individual clinician factors.
- Context—decision-making in EGS.

#### Inclusion criteria

Any studies reporting on clinician factors (as detailed in introduction) and decision-making in EGS will be eligible. Only studies undertaken in adult populations (patients aged 18 and over) will be eligible for inclusion. We will search for peer-reviewed published articles and selected grey literature resources (conference abstracts, theses and pre-publication texts) provided sufficient methodology is available. The inclusion of selected grey literature aims to reduce publication bias and ensure a comprehensive review of available literature.

This review will consider quantitative, qualitative and mixed-methods study designs. There will be no time limit applied to the studies when searching. There will be no language limit applied to the search. If non-English language articles are found, we will use Google Translate to assess the abstract and, if seemingly relevant, will use a professional translation service to translate the articles in question.

#### Exclusion criteria

Studies in paediatric populations (patients aged under 18 years old) will be excluded; this is primarily due to differences in decision-making between adult and paediatric populations. In paediatric patients the patient primary decision makers are often parents/caregivers on behalf of their children, and this duly impacts the decision-making process.

Studies examining elective surgeries only will also be excluded. This review is focusing on decision making in the high acuity and time-pressured environment of EGS. The inclusion of elective surgery is out with the scope of this review. Studies examining solely trauma surgery or non-general surgical specialities will be excluded.

Selected grey literature, such as editorials and book chapters, will be excluded as they do not provide primary research data to analyse.

### Search strategy

Here we present the full electronic search strategy for MEDLINE (via OVID), including any limits used, such that the search could be repeated.

Full search strategy for MEDLINE detailed in online supplemental appendix 1.

### Information sources

Databases to be searched: MEDLINE (via OVID), EMBASE (via OVID), Cochrane Database of Systematic Reviews (CDSR), Cochrane Central Register of Controlled Trials (CENTRAL) and Cumulative Index to Nursing and Allied Health Literature (CINAHL via EBSCOhost). The search will be adapted for each included database and/or information source.

The reviewers will also check the reference list of eligible studies to look for other potentially appropriate studies for inclusion, a technique known as snowballing. We will conduct an online search to assess for relevant conference abstracts and theses.

### Selection of sources of evidence and data charting process

Articles identified in searching will have their citation details imported into Covidence. Covidence is an online, collaborative software platform which will provide a structured framework to this review and allow for the collaboration of multiple reviewers.^19^ Covidence will be used to perform deduplication of articles.

Screening will be completed by two independent reviewers (EG, RB) who will assess titles and abstracts of articles against the pre-determined inclusion and exclusion criteria to determine which abstracts will be retrieved in full. Full text articles will be reviewed against inclusion criteria by two independent reviewers (EG, RB). If any disputes arise during the screening and selection process, a third independent reviewer (RJ-C) will decide any disputes.

The results of the search and the study inclusion process will be reported in full in the final scoping review and presented in a PRISMA flow diagram.^17^

### Data extraction

Data will be extracted from sources of evidence included in the scoping review by two independent reviewers (EG, RB) using a data extraction form created by the reviewer (EG). Data extracted will include study bibliographic details, specific details about study design, clinician factors studied, data collection measures used and the key results.

A draft data extraction form is included (online supplemental appendix 2).

### Data analysis and presentation

The extracted data will be displayed in a table form. A narrative summary will be provided which will aim to map key concepts, describe study characteristics and identify gaps in the literature. The review does not aim to perform a risk of bias assessment, as this is typically not undertaken during a scoping review. The aim of this scoping review, in alignment with the intention of scoping reviews in general, is not to seek the best available evidence, but instead provide a comprehensive description of the available evidence related to the question of interest.

### Patient and public involvement

Patients and the public were not involved in the design of this scoping review protocol.

## Ethics and dissemination

Ethics approval is not required for this scoping review, as this review will use publicly available data. Results will be disseminated via presentation at surgical conferences and submission for publication at peer-reviewed journals.

## Supplementary material

10.1136/bmjopen-2026-116205 online supplemental file 1
