# Supplementary material for: Clinician factors influencing decision-making in emergency general surgery (EGS): a scoping review protocol
Source: BMJ Open. 2026 Apr 15;16(4):e116205. doi: 10.1136/bmjopen-2026-116205 (PMC13084799; doi:10.1136/bmjopen-2026-116205)

Appendices

Appendix 1 – Full Search Strategy for Ovid MEDLINE

Ovid MEDLINE(R) ALL <1946 to December 03, 2025>

1. General Surgery/
2. Acute care surgery/ or "acute care surg*".ti,ab,kf.
3. Laparotomy/
4. 1 or 3
5. Emergency Treatment/
6. Emergencies/
7. 5 or 6
8. ((emergency or emergencies or emergent or urgent or expedite*) adj3 (surger* or surgical* or operation or operations or operative or procedure or procedures or reoperat* or laparoscop* or laparotom* or resection* or context)).ti,ab,kf.
9. 4 and 7
10. 2 or 8 or 9
11. Clinical Decision-Making/
12. Decision Making, Shared/
13. Decision Making/
14. Judgment/
15. Uncertainty/
16. decision-making.ti,ab,kf.
17. clinical judgement*.ti,ab,kf.
18. uncertain*.ti,ab,kf.
19. treatment choice*.ti,ab,kf.
20. (decision* adj3 (tool* or aid or aide or aids or (support* adj3 technique*))).ti,ab,kf.
21. deliberation*.ti,ab,kf.
22. 11 or 12 or 13 or 14 or 15 or 16 or 17 or 18 or 19 or 20 or 21
23. 10 and 22

Appendix 2 – Data Extraction Form


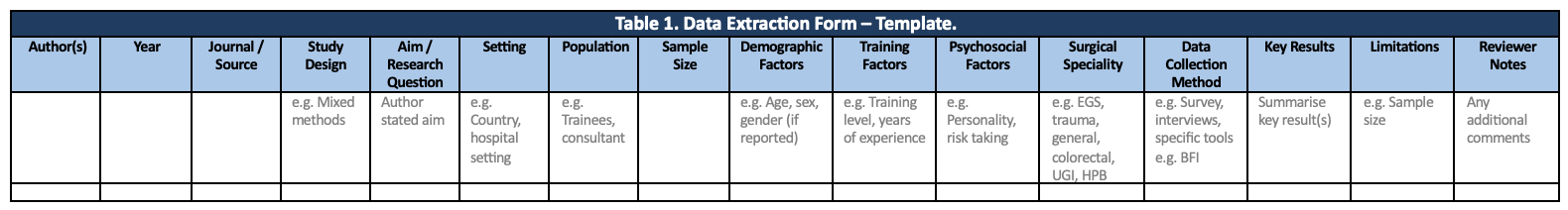


Appendix 3 – PRISMA P Checklist(18)


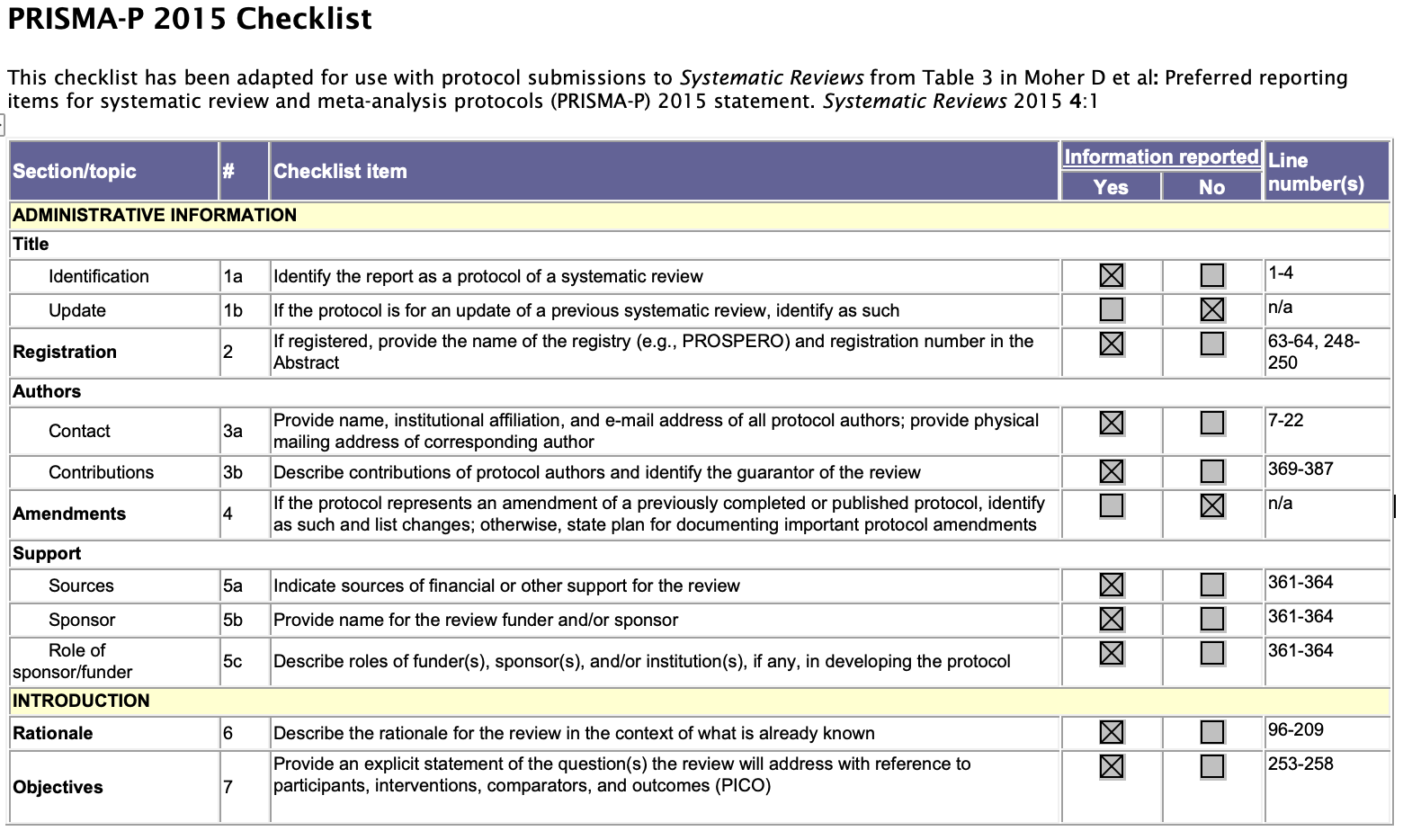

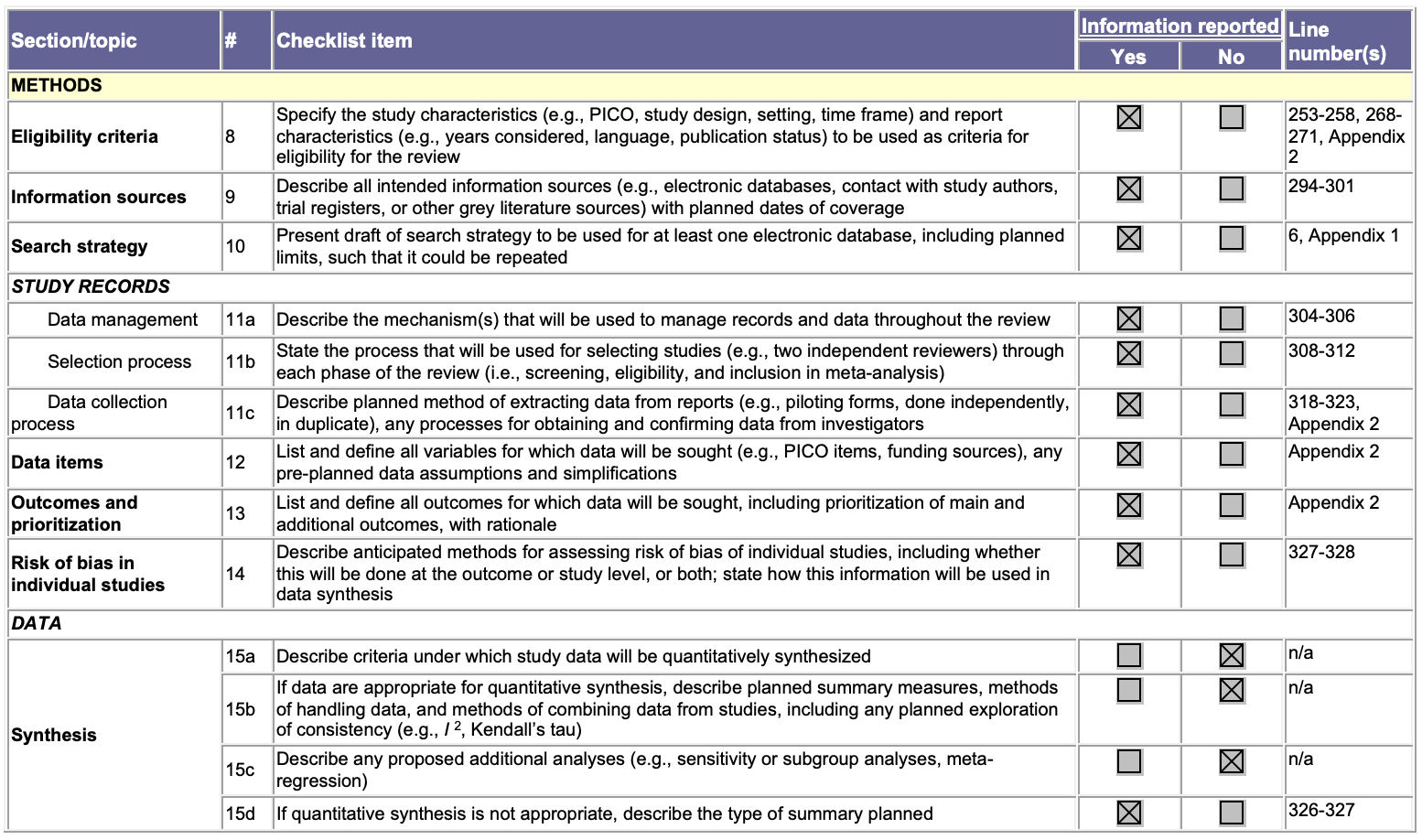

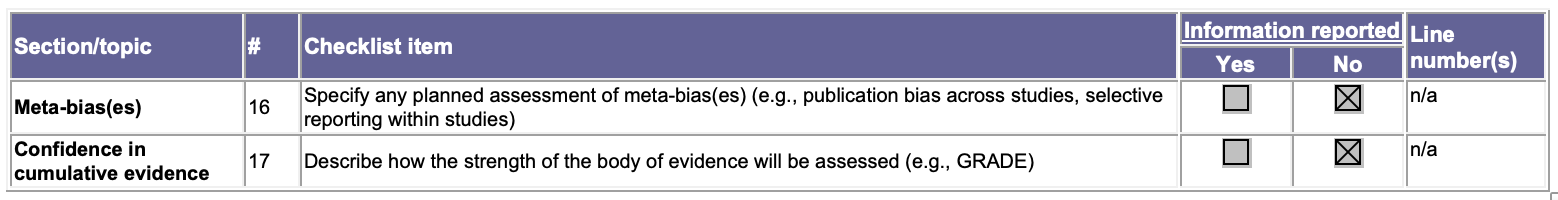

Supplement: online supplemental file 1 [file bmjopen-16-4-s001.docx]
