# Supplementary material for: Clinician factors influencing decision-making in emergency general surgery (EGS): a scoping review protocol
Source: BMJ Open. 2026 Apr 15;16(4):e116205. doi: 10.1136/bmjopen-2026-116205 (PMC13084799; doi:10.1136/bmjopen-2026-116205)
Supplement: Supplementary file 2 [file bmjopen-2026-116205.reviewer_comments.pdf]

PEER REVIEW HISTORY

BMJ Open publishes all reviews undertaken for accepted manuscripts. Reviewers are asked to complete a checklist review form and are provided with free text boxes to elaborate on their assessment. These free text comments are reproduced below.

ARTICLE DETAILS

Title (Provisional)

Clinician Factors Influencing Decision-Making in Emergency General Surgery (EGS): A Scoping Review Protocol

Authors

Groundwater, Ellen; Bisset, Carly Nichola; Bradley, Rebecca; John-Charles, Rachel; Maeda, Yasuko; McKee, Tracey; Moug, Susan Joan

---

VERSION 1 - REVIEW

**Reviewer**	**1**
**Name**	**Leslie, Kate**
**Affiliation**	**The Royal Melbourne Hospital**
**Date**	**21-Feb-2026**
**COI**	**None**

The manuscript submitted to BMJ Open on January 2nd 2026, was received by me on February 20th 2026 and was reviewed on February 22nd 2026.

This manuscript presents a protocol for a scoping review about clinician factors that may influence decision-making in emergency general surgery patients.

The topic is interesting and important, the protocol is clear and the manuscript is well-written.

The protocol has been registered on the Open Science Framework site (the manuscript is inconsistent on this point).

The appropriate PRISMA checklist is attached.

It is not completely clear what types of decisions are of interest or likely to be included (e.g. proceed to surgery, type of operation, type of anaesthetic or pain management technique). Perhaps this could be added to the introduction?

It is likely that clinician sex (i.e. male, female) will be reported rather than gender identity (i.e. man, woman, non-binary etc.). Are you collecting both?

VERSION 1 - AUTHOR RESPONSE

Reviewer 1 Comments

Comment: The protocol has been registered on the Open Science Framework site but the manuscript is inconsistent on this point

Response:

We thank the reviewer for highlighting this inconsistency. The manuscript has been revised to ensure consistent reporting of the prospective registration on the Open Science Framework.

(Page 2, line 63-64 and Page 6, lines 248-250)

Comment: It is not completely clear what types of decisions are of interest or likely to be included

Response:

We thank the reviewer for this helpful comment. Additional clarification has been added to the introduction to better define the types of clinical decisions which may be captured in the planned scoping review.

(Page 3, lines 114-118)

Comment: It is likely that clinician sex will be reported rather than gender identity. Are you collecting both?

Response:

The manuscript and data extraction form have been revised to clarify that clinician sex and/or gender will be extracted and reported depending on how these variables are reported in the included studies.

(Page 4, line 179 and Appendix 2)
